# Photo-induced surface-enhanced Raman spectroscopy from a diphenylalanine peptide nanotube-metal nanoparticle template

**DOI:** 10.1038/s41598-018-22269-x

**Published:** 2018-03-01

**Authors:** Sawsan Almohammed, Fengyuan Zhang, Brian J. Rodriguez, James H. Rice

**Affiliations:** 1 School of Physics, University College Dublin, Belfield, Dublin 4 Ireland; 2 Conway Institute of Biomolecular and Biomedical Research, University College Dublin, Belfield, Dublin 4 Ireland

## Abstract

UV irradiation of aligned diphenylalanine peptide nanotubes (FF-PNTs) decorated with plasmonic silver nanoparticles (Ag NPs) enables photo-induced surface-enhanced Raman spectroscopy. UV-induced charge transfer facilitates a chemical enhancement that provides up to a 10-fold increase in surface-enhanced Raman intensity and allows the detection of a wide range of small molecules and low Raman cross-section molecules at concentrations as low as 10^–13^ M. The aligned FF-PNT/Ag NP template further prevents photodegradation of the molecules under investigation. Our results demonstrate that FF-PNTs can be used as an alternative material to semiconductors such as titanium dioxide for photo-induced surface-enhanced Raman spectroscopy applications.

## Introduction

Surface-enhanced Raman spectroscopy (SERS) is a subject of considerable research interest due to its ultrasensitive detection, analysis, and imaging applications^1^. There are two SERS enhancement mechanisms: electromagnetic and chemical. The electromagnetic enhancement mechanism is related to the surface topography and the wavelength-dependent plasmonic properties of the metal surface^2,3^. The conduction electrons of the metal can be stimulated by the incident electric field in collective oscillations known as localized surface plasmon resonances (SPRs) that localize the electromagnetic field at sub-wavelength scales when excited by light, creating electromagnetic hot spots. Raman scattering from molecules positioned in these hot spots can be enhanced by factors of ~10^10^–10^14 ^^2–4^. Chemical enhancement results from the chemical interaction between molecules and a surface and is mediated through charge transfer. The charge transfer process can be summarized in four steps: (i) excitation of an electron into a hot electron state, (ii) transfer of the hot electron into the lowest unoccupied molecular orbital of the molecule, (iii) transfer of the hot electron from the lowest unoccupied molecular orbital back to the metal, and (iv) the return of the electron to its initial state and Stokes photon creation^5–8^. Many substrate designs have been developed with the aim of increasing the Raman scattering signal through the chemical enhancement mechanism, including the use of semiconductor materials, such as titanium dioxide (TiO_2_) and carbon nanotubes (CNTs)^9–16^. However, chemical enhancement factors are typically lower (~ 10^2^) than those reported for plasmon-active metal based substrates. Combining plasmon-active metallic NPs and semiconducting materials allows both mechanisms to be exploited simultaneously^9–12^ and has resulted in improved sensitivity and detection, allowing detection down to the single molecule level^12–16^.

A recent study combining plasmon-active silver NPs with a film of semiconducting TiO_2_, which was irradiated using a UV light source prior to Raman measurements, demonstrated that above bandgap excitation enabled additional Raman enhancement beyond that expected from NPs alone^17^. The authors assigned this chemical enhancement to the transfer of photogenerated electrons from the sample to the probe molecule^17^. Self-assembly of organic semiconductor peptide-based materials has emerged as a new approach for the fabrication of SERS substrates, offering an alternative to more traditionally used semiconductor materials such as TiO_2_^2,7,18–24^. Diphenylalanine peptide nanotubes (FF-PNTs) are particularly interesting due to their high thermal stability^25–32^, high stiffness and elastic modulus^31,32^, ease of preparation^25–28^, biocompatibility^33^, piezoelectricity^34^, ability to bind with metal ion residues at specific locations^35–38^, and wide bandgap (*E*_*g*_ ~ 4.6 eV)^39–41^. FF-PNTs are almost transparent to visible (380–750 nm) and near-UV (300–380 nm) light, and they strongly absorb light in the middle-UV spectral region (200–300 nm)^41–44^, making them suitable for a variety of light harvesting and catalytic applications^45–47^. FF-PNTs have been shown to enhance the photo luminescent properties of photosensitizer molecules via a cascaded energy transfer process^44^ and to mediate the transfer the energy from photo-excited molecules or chromophores to catalytic centers^44,48^. Such charge transfer processes in FF-PNTs have been reported to be strongly affected by structural changes, heat, and UV irridiation^39–41^. The described properties make FF-PNTs an attractive alternative material to more traditional semiconductors such as TiO_2_ or CNTs for SERS-based applications^9,10,14–16^.

Despite the fact that many substrate designs have been developed to increase Raman intensity, there are very few studies in the literature that have used FF-PNTs as a template to support SERS active substrates^49–51^. It has been previously reported that aligned FF-PNT templates formed with plasmon-active Ag NPs enabled SERS detection of molecules^49,50^. Here, we report UV-enhanced SERS from Ag NPs on aligned, semiconducting FF-PNTs. The aligned FF-PNT/Ag NP template enables a large signal enhancement for a wide range of molecules while preventing photodegradation typically observed when Ag NPs on Si are used.

## Results and Discussion

**Figure 1:**
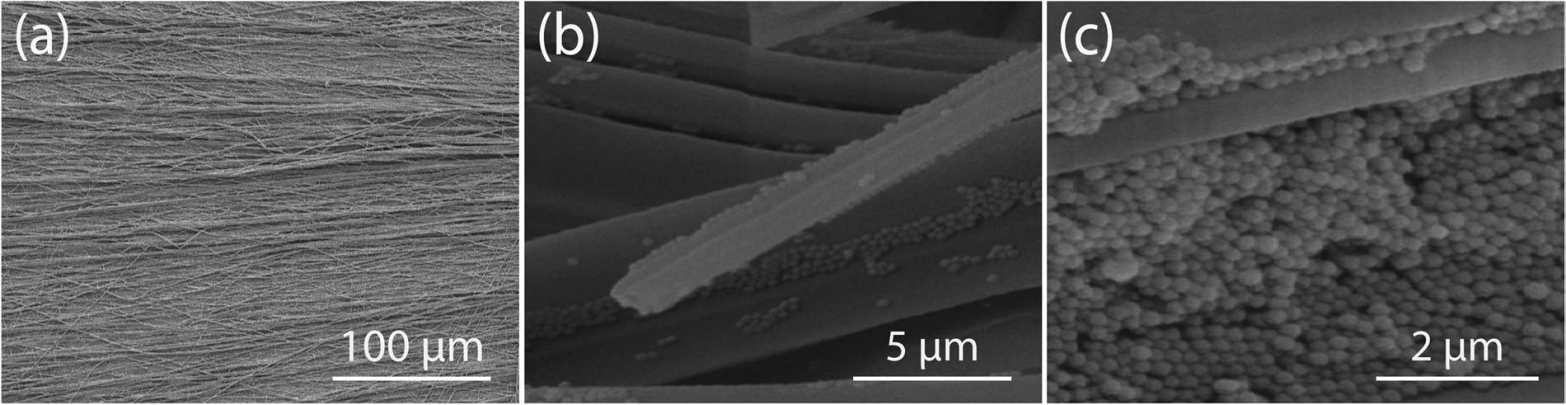
SEM images of an aligned (left to right) FF-PNT/Ag NP template.

Aligned FF-PNTs were formed with Ag NPs following a previously reported procedure (see supplementary information (Fig. S1))^49^. Scanning electron microscopy (SEM) images of the resulting aligned FF-PNT/Ag NP template (Fig. 1) shows aligned FF-PNTs (with an average diameter of 3.2 ± 1.3 μm determined from n = 30 FF-PNTs) with Ag NPs clusters. The arrangement of FF-PNTs likely promotes the confinement of Ag NPs.

**Figure 2:**
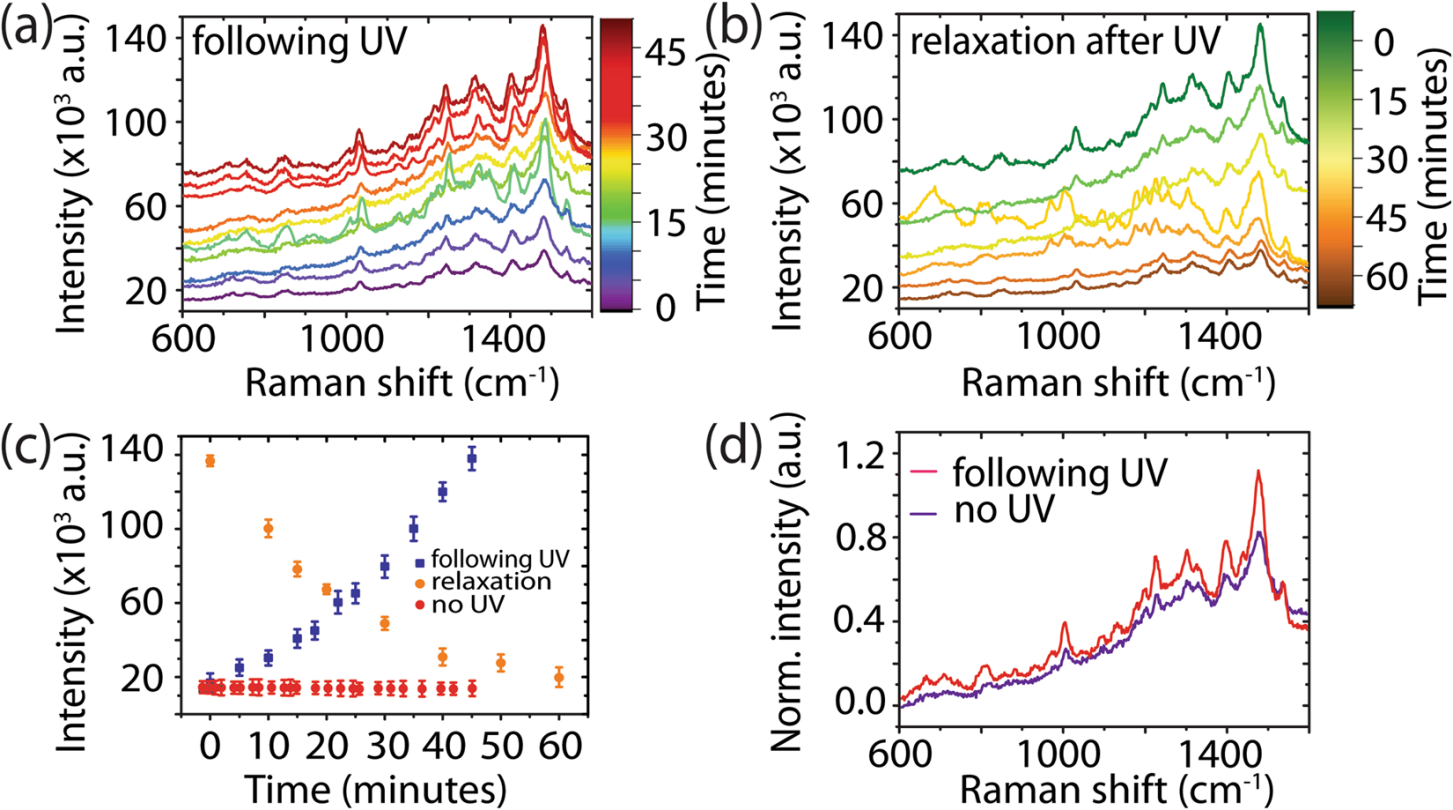
(**a**) SERRS measurements of the aligned FF-PNT/Ag NP template with TMPyP following UV irradiation. (**b**) SERRS measurements recorded following 45 minutes of UV irradiation, showing the relaxation of the signal. (**c**) Plots of intensity vs. time following 5 minute irradiation steps (blue), after turning the UV lamp off after 45 minutes of total UV irradiation (orange), and in the absence of UV irradiation (red). (**d**) A comparison of normalized SERRS spectra with (red) and without (purple) UV irradiation.

Following the deposition of meso-tetra (N-methyl-4-pyridyl) porphine tetrachloride (TMPyP)10^–6^ M on the template, Raman measurements were performed (Fig. 2a (0 minutes)). TMPyP possesses an optical absorption Q band (Fig. S2) in resonance with the Raman laser excitation wavelength, resulting in surface-enhanced resonance Raman spectroscopy (SERRS). The sample was then irradiated for 5 minutes using light with an energy (4.8 eV) greater than the bandgap of the FF-PNTs (*E*_*g*_ ~ 4.6 eV)^39–41^. With the UV lamp off, Raman measurements were again performed and the process was repeated until the total irradiation time was 45 minutes. A 7-fold increase in SERRS intensity following UV irradiation was seen in comparison to non-irradiated substrates (Fig. 2a–d). The SERRS spectra before and after UV irradiation possess Raman spectral features in agreement with those reported in the literature for TMPyP, e.g., C-pyrrole bending at 1249 cm^−1^, C-C stretching at 1451 and 1557 cm^−1^, and pyrrole bending at 1639 cm^−1 ^^52,53^. Plotting SERRS intensity at 1448 cm^−1^ vs. UV irradiation time (Fig. 2c) shows that following 45 minutes of UV irradiation in total, the SERRS intensity rose from 19 × 10^3^ to 142 × 10^3^ cm^−1^. Over the course of 60 minutes after the UV lamp was turned off, the SERRS intensity decayed to the original intensity, demonstrating that the process is reversible. Without UV irradiation (Fig. 2c (red)), no changes in the SERRS signal was observed over time, confirming that UV irradiation is responsible for the increase the SERRS signal from TMPyP. Normalized SE(R)RS spectra with (red) and without UV irradiation (purple) are shown in Fig. 2d. No photodegradation was observed in the absence of UV or following 45 minutes of UV irradiation. In contrast, SERRS measurements (Fig. S3) performed on a Si substrate, with *E*_*g*_ = 1.1 eV^54,55^, having only Ag NPs and TMPyP (i.e., no FF-PNTs) revealed significant photodegradation of the probe molecule, confirming that the presence of FF-PNTs aids in the prevention of photodegradation of the probe molecule, perhaps as a result of the thermal and pyroelectric properties of FF-PNTs^56^. It should be emphasized that the increased SERRS intensity from TMPyP on aligned FF-PNT/Ag NP templates following UV irradiation occurs when using a 254 nm lamp and not with a 365 nm (3.39 eV) lamp (Fig. S4). Moreover, comparable enhancements were also observed from TMPyP on aligned FF-PNT/Au NP templates (Fig. S5), demonstrating that both chemical and electromagnetic enhancement is present.

**Figure 3:**
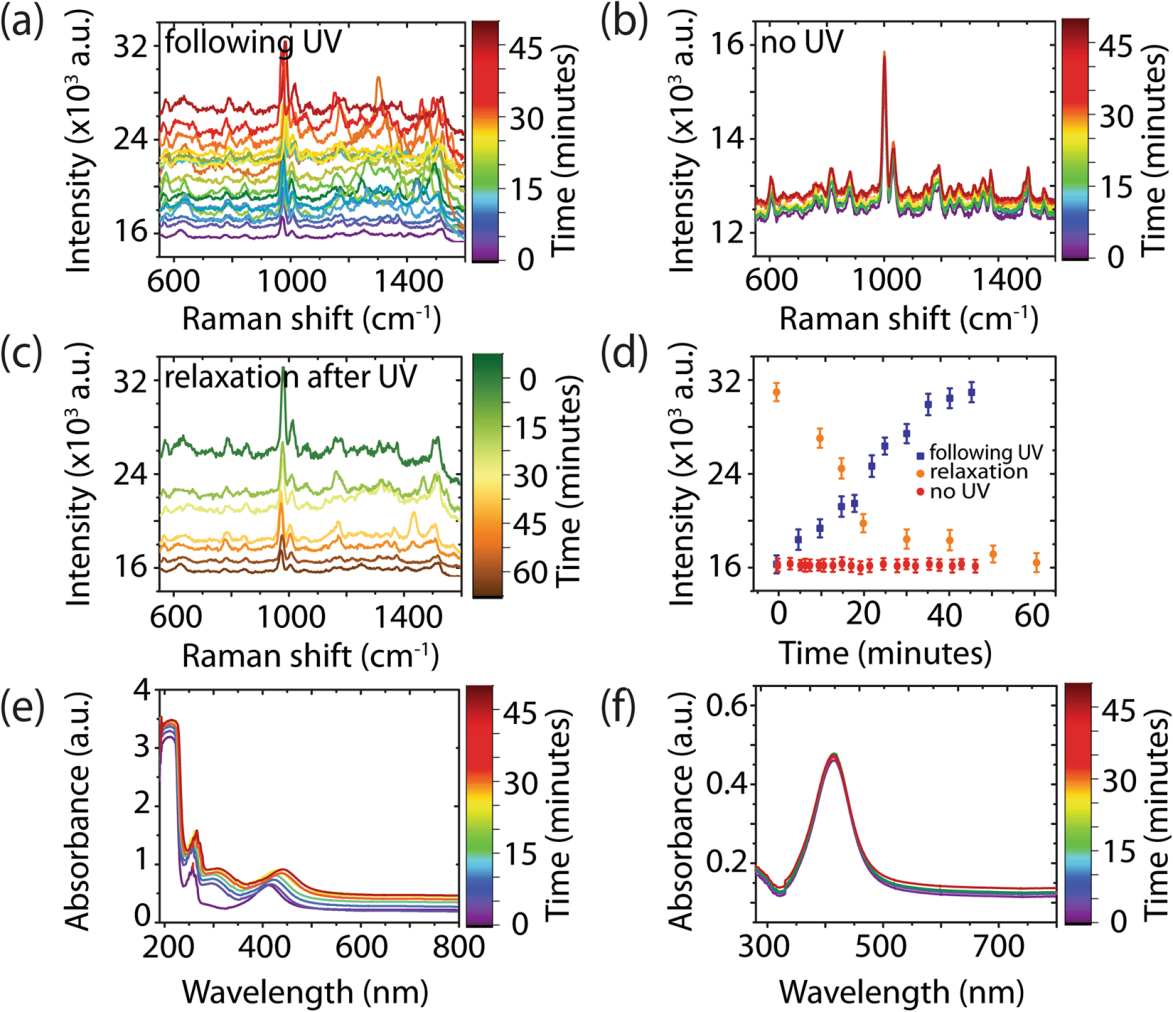
SERS measurements of the aligned FF-PNT/Ag NP template without a probe molecule present (**a**) following UV irradiation in 5 minute increments, (**b**) in the absence of UV irradiation over a 45 minute period, and (**c**) over the course of 60 minutes following the removal of UV irradiation. (**d**) Plot of intensity vs. time for the Raman band at 1003 cm^−1^ following UV irradiation (blue), without UV (red), and after 45 minutes of UV irradiation (orange). The inset shows the normalized spectra for the aligned FF-PNT/Ag NP template without UV irradiation and after relaxation from UV. (**e**) Absorption spectra of FF-PNT/Ag NP solution before and after UV irradiation. (**f**) The absorption spectra of Ag NPs alone before and after UV irradiation.

To understand the origin of the observed increase in intensity with UV irradiation, Raman was performed on the aligned FF-PNT/Ag NP template without a probe molecule (Fig. 3). The SERS spectrum of the template is assigned to arise from FF-PNTs with peak positions and relative intensities in agreement with those reported in the literature, e.g., an aromatic ring breathing mode at 1002 cm^−1^ and a phenyl vibrational band at 1603 cm^−1 ^^57^. UV irradiation increases the SERS intensity arising from the aligned FF-PNT/Ag NP template (Fig. 3a). The SERS spectra show an increase in Raman intensity from 16 × 10^3^ to 31.8 × 10^3^ (2-fold increase) following a total irradiation time of 45 minutes in 5 minute increments. Inspection of the spectral features shows that random fluctuations in SERS peak intensities are present in the SERS spectra. Such blinking may arise from UV-induced charge transfer processes between FF-PNTs and Ag NPs^32,57,58^. In contrast, the SERS spectra for the FF-PNT/Ag NP template without UV irradiation (Fig. 3b) shows no changes in SERS intensity or peak positions. Following removal of UV irradiation, the SERS intensity of the template drops over time (Fig. 3c–d). The SERS intensity relaxes to pre-irradiation levels over the course of 60 minutes.

The absence of photodegradation of the FF-PNT/Ag NP solution was confirmed through Fourier Transform Infrared Spectroscopy (FTIR) (Fig. S6). The spectral features are similar to that reported earlier for diphenylalanine nanotubes; the broad nature of the amide I band at 1630 cm^−1^ corresponds to β-sheet structure during peptide nanotube formation^59^. The FTIR data show no changes in spectral features following 45 minutes of UV irradiation. Additionally, optical images of the aligned FF-PNT/Ag NP template (Fig. S7) after UV irradiation for 20, 30, and 45 minutes show no apparent deterioration of the template, confirming the templates are stable under UV irradiation.

To demonstrate the charge transfer interaction between FF-PNTs and Ag NPs, the absorption spectra of FF-PNT and Ag NP solutions before and after UV irradiation were measured. For Ag NPs in the absence of FF-PNTs (Fig. 3e) a broad SPR band with a peak at 418 nm (full-width at half maximum of 45 nm) was observed. The FF-PNT solution was transparent over the visible range from 300 to 750 nm (Fig. S8). The characteristic absorption bands of FF-PNTs are located at 222 nm and several peaks are centered at 255 nm. These peaks are assigned to electron transitions in the phenyl groups of the FF-PNT^44,48^. Comparison of the absorption spectra for Ag NPs recorded in the presence of FF-PNTs relative to when FF-PNTs are absent (Figs 3e and S8) shows that the presence of FF-PNTs creates a narrowing in the Ag NP SPR band and a red shift in peak position. This potentially arises from FF-PNT/Ag NP interactions mediated by the presence of amino acid functional groups on the surface of FF-PNTs supporting binding interacting with the Ag NPs. This is in line with previous reports that carboxylate groups bind to metal NPs, which can result in a red shift in the SPR absorption band of the NPs^35–38^. Studies of aligned FF-PNTs (as are also used here) with increasing UV excitation intensity showed that the density of the photogenerated carriers increases and formed a built-in electric field which is opposite to the intrinsic surface plasmon field^45–47^. Potentially, this effect can alter the plasmon energy of Ag NPs present on the FF-PNTs. The red shift in the optical absorption spectra with increasing UV irradiation may potentially arise from this effect.

The absorption spectra of the aligned FF-PNT/Ag NP template were recorded as before, when the UV lamp was off, following UV irradiation in 5 minute increments up to a total irradiation time of 45 minutes (Fig. 3e). A significant red shift of the Ag NP SPR band (430 to 450 nm) is observed upon irradiation, which then blue shifts back to 428 nm following the removal of the UV irradiation source. The degree of red shift was proportional to the UV irradiation time (Fig. 3e inset). This red shift is accompanied by a wider SPR bandwidth (a full-width at half maximum increase from 30 to 42 nm upon UV irradiation). The red shift can be explained by an increase in Ag NP electron density during irradiation that changes the reflective index of the Ag NPs^2,7,18–22,60,61^. Following the approach reported by Mulvaney *et al*.^62^, we estimate the injected electron density (Δ*N*/*N*) on the Ag NPs following UV irradiation using equation 1:

1 $$\frac{{\rm{\Delta }}N}{N}=2{\rm{\Delta }}{\rm{\lambda }}/{{\rm{\lambda }}}_{0}$$

where Δλ is the measured wavelength shift (~20 nm) and λ_0_ is the initial Ag NPs plasmon peak position for the FF-PNTs (~430 nm). From Fig. 3e, we can calculate Δ*N*/*N* ~ 9%, a value higher than reported for SERS experiments using TiO_2_ and metal NPs (Δ*N*/*N* ~ 4%)^17^. The injected charge likely leads to shifts in the Fermi level of the nanoparticle to more negative potentials^17^. The presence of these excess electrons on the Ag NPs enhances the Raman signal intensity chemically^17^. When UV irradiation absorbance experiments were performed on Ag NPs, no changes in the SPR peak position were seen (Fig. 3g), confirming a role of FF-PNTs in the charge transfer process.

**Figure 4:**
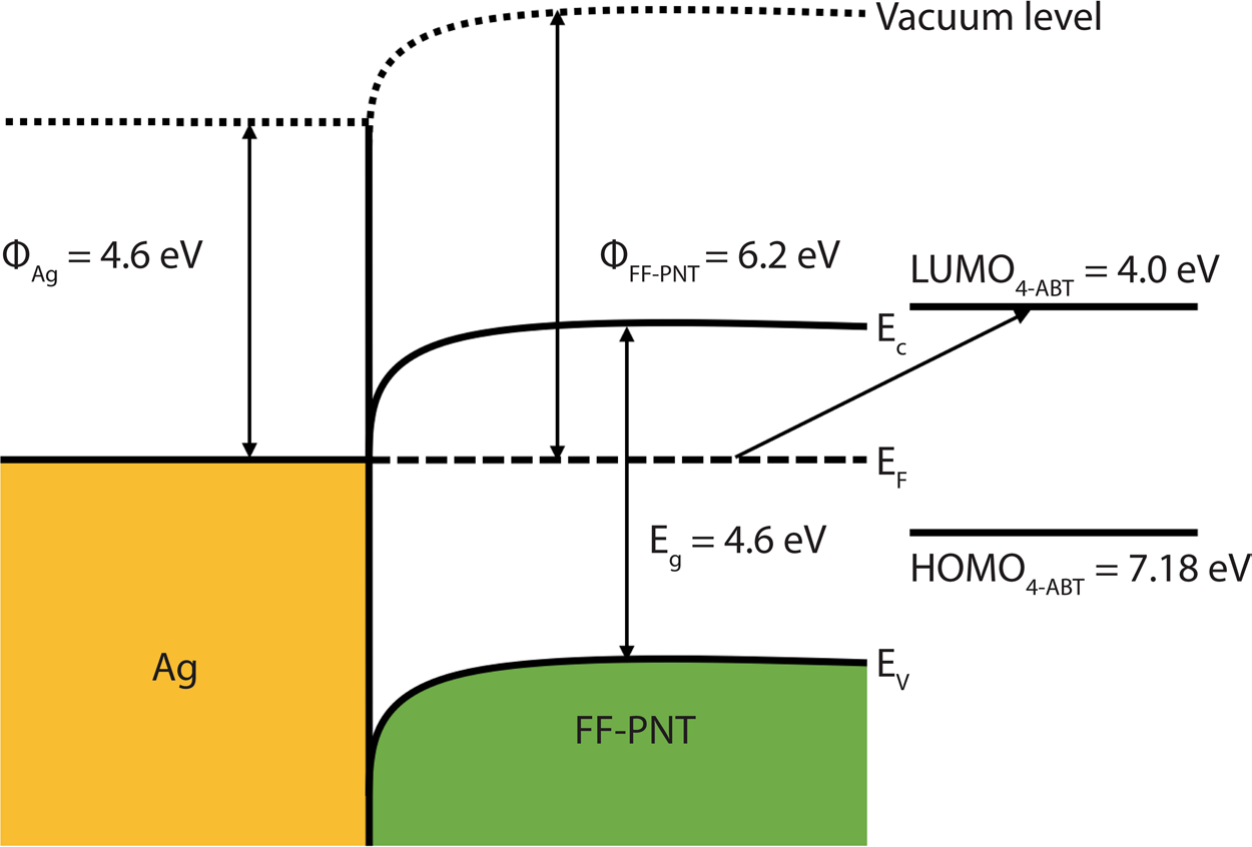
Proposed band structure of the Ag-FF-PNT junction where Φ is the workfunction, E_f_ is the Fermi level, E_c_ is the conduction band, and E_v_ is the valance band. The highest occupied molecular orbital (HOMO) of 4-ABT is located 7.18 eV below the vacuum level while the lowest unoccupied molecular orbital (LUMO) is located 4.0 eV below the vacuum level.

Photogenerated electrons are thus transferred from the FF-PNTs to the Ag NPs as the two systems undergo charge equilibration^17,39–41,45–47^. To further understand the charge transfer process between FF-PNTs and Ag NPs, we have estimated the workfunction of FF-PNTs based on previously reported Kelvin probe force microscopy (KPFM) data^63^. In KPFM, the contact potential difference (CPD) is defined by equation 2:

2 $${\rm{CPD}}=\frac{{{\rm{\varphi }}}_{{\rm{tip}}}-{{\rm{\varphi }}}_{{\rm{sample}}}}{{\rm{q}}}$$

where ϕ_tip_ and ϕ_sample_ are the workfunction of the tip and sample, respectively, and q is the elementary charge^63^. The difference in CPD between FF-PNTs and SiO_2_ was reported to be ΔCPD = ~1.5 V^63^. From equation 2 and assuming the workfunction of SiO_2_ is 4.7 eV^54,55^ the workfunction of FF-PNTs can be estimated as 6.2 eV. From this value, the movement of electrons from the FF-PNTs to the Ag NPs is possible, as illustrated in a proposed metal-semiconductor junction band structure for Ag and FF-PNT (Fig. 4). KPFM data obtained from an aligned FF-PNT sample collaborates this finding; the surface potential of FF-PNTs was determined to be ~6.3 eV (Fig. S9). As further evidence that charge transfer from FF-PNTs can occur under UV irradiation, we note that when FF-PNTs are irradiated with 254 nm UV light in the presence of aqueous AgNO_3_, Ag NPs form at the surface of the FF-PNTs (Fig. S10), which are also SERS-active (Fig. S11). The reduction of Ag^+^ to Ag^0^ requires electrons and NPs do not form in the absence of UV irradiation. The presence of photogenerated charge is further supported by photoluminescence measurements. Photoluminescence was measured from an aligned FF-PNT/Ag NP template during UV irradiation up to 45 minutes in 5 minute increments (Fig. S12). The intensity of the fluorescence peak at 300 nm arising from a n–p* transition in the FF-PNTs decreased with increasing UV irradiation time. This quenching in emission intensity is a strong indication that charge transfer processes take place^41–44^.

**Figure 5:**
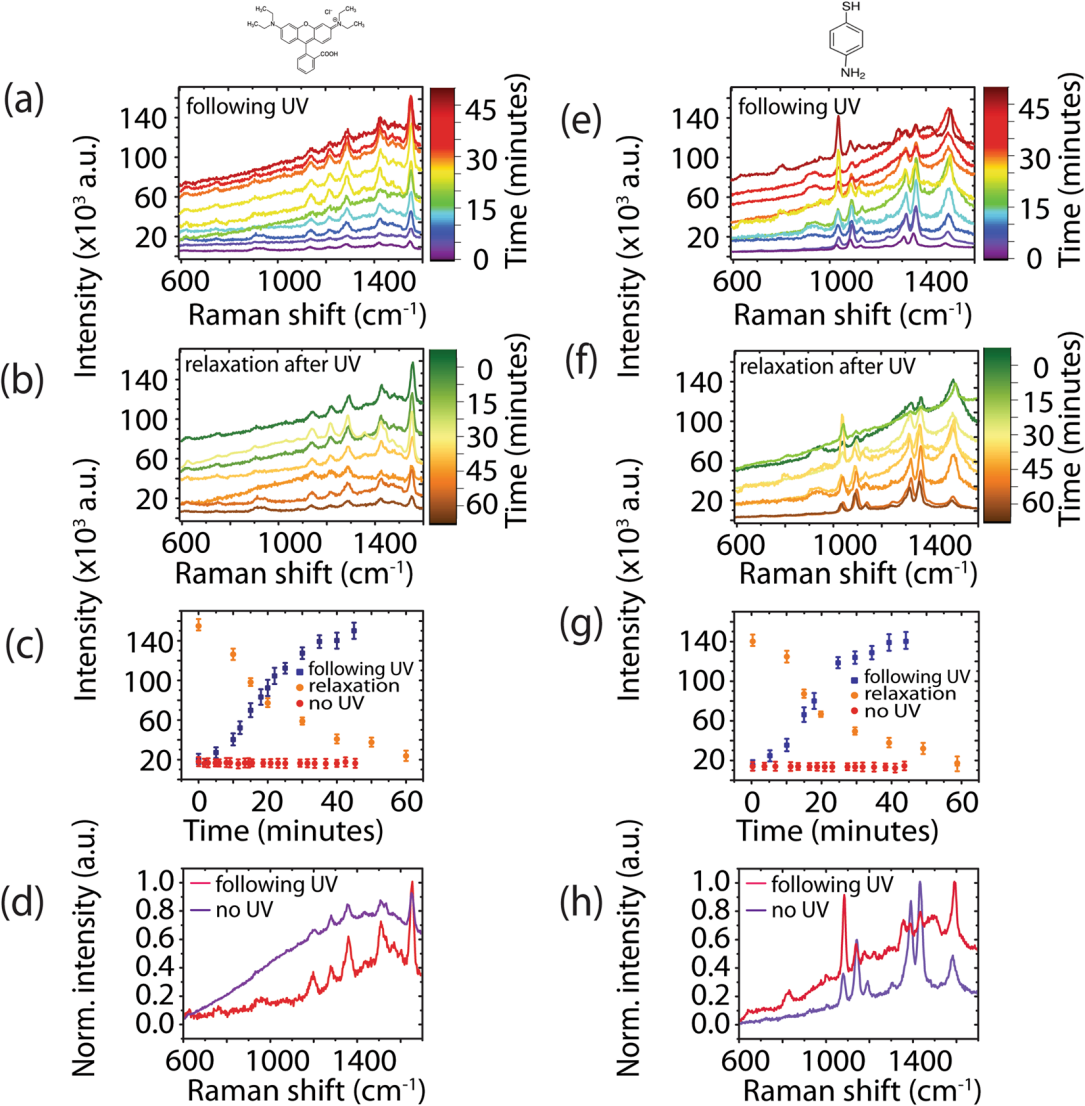
SE(R)RS measurements of the aligned FF-PNT/Ag NP template with the probe molecules (**a**–**d**) RhB and (**e**–**h**) 4-ABT (**a**,**e**) following 254 nm UV irradiation in 5 minute increments and (**b**,**f**) after the final irradiation step (45 minutes total). (**c**,**g**) Plots of intensity vs. time following UV irradiation (blue), after turning the UV lamp off (orange), and in the absence of UV irradiation (red). (**d**,**h**) A comparison of SE(R)RS spectra with (red) and without UV irradiation (purple).

A second probe molecule, 10^–6^ M rhodamine B (RhB), was also investigated (Fig. 5a–d). The UV irradiation and Raman measurement procedure was performed as before for TMPyP. Following 45 minutes of UV irradiation, the SERRS intensity increased from 19.8 × 10^3^ to 160 × 10^3^ (8-fold increase). After turning off the UV lamp permanently, the SERRS signal of RhB, like TMPyP, reduced from 160 × 10^3^ to 20 × 10^3^ over the course of 60 minutes (Fig. 5b–c). RhB molecules possess a strong absorption band in resonance with the excitation laser (Fig. S2). In addition, the fluorescence emission wavelength has a small Stokes shift resulting in the fluorescence overlapping with the Raman wavelength window. Exposing the substrate to UV irradiation appears to quench the fluorescence background (in line with the presence of charge transfer processes)^41–44^, making the Raman peaks of RhB more clearly distinguishable, in comparison to a non-UV irradiated SERRS spectrum (Fig. 5d). Studies have shown that plasmonic nanomaterials and semiconductor materials can quench fluorescence of aromatic compounds either via electron or energy transfer^2,64^. The SERRS spectrum shows bands at 1649, 1593, 1509, 1359, and 1280 cm^−1^, which are assigned to the probe molecule, in line with literature^65^. These bands are present following UV irradiation, indicating that no photodegradation has occurred, similar to the results observed when using TMPyP following UV irradiation.

A third molecule, 4-ABT was also investigated using the same procedure (Fig. 5e–h). This molecule possesses an absorption band in the UV region (<350 nm) (Fig. S2). SERS measurements were performed using a 532 nm excitation laser, resulting in an absence of Raman resonance enhancement. The SERS spectrum recorded for 4-ABT prior to UV irradiation is in line with what has been reported previously, showing Raman bands at 1590, 1432, 1390, 1144, and 1076 cm^−1 ^^66,67^. This SERS spectrum is assigned to the presence of dimers of 4-ABT^67^. Under UV irradiation for a period from 5 minutes to 45 minutes as before, a 10-fold increase in Raman intensity was observed from 18.6 × 10^3^ before irradiation to 140 × 10^3^ after 45 minutes of total irradiation (Fig. 5g). Relaxation back to 18.6 × 10^3^ was observed following the removal of UV irradiation (Fig. 5g). The relaxation in SERS signal intensity was accompanied by changes in the spectral profile. Before UV irradiation, the SERS spectrum showed five intense bands at 1590, 1432, 1390, 1144, and 1076 cm^−1^. After UV irradiation, these bands increased in intensity (Fig. 5h). These changes in relative intensities are accompanied by the appearance (with increasing UV irradiation) of a new band at 1335 cm^−1^ (Fig. 5h), which has been assigned to the presence of nitrophenol^67,68^. It is reported that nitrophenol is formed from 4-ABT through a charge transfer-based reaction under UV illumination^67,68^. The strong SERS enhancement seen in 4-ABT can also be understood as vibronic (Herzberg-Teller) coupling of the plasmon resonance with charge transfer resonances in the molecule-substrate system^69^. The SERS spectra for 4-ABT (Fig. 5e) has peaks at 1432, 1390, and 1144 cm^−1^, which have been shown to be sensitive to charge transfer effects^69^. In Fig. 5e, the intensity of these bands are enhanced with UV irradiation of the aligned FF-PNT/Ag NP template, indicating the possibility of charge transfer between the FF-PNT/Ag NP template and the probe molecule, 4-ABT. The highest occupied molecular orbital (HOMO) of 4-ABT is located 7.18 eV below the vacuum level while the lowest unoccupied molecular orbital (LUMO) is located 4.0 eV below the vacuum level^69^. The difference between the Fermi level of the aligned FF-PNTs (6.2 eV below the vacuum level, shown schematically in Fig. 4) and the LUMO is 2.2 eV, which is less than the laser excitation (532 nm; 2.3 eV). Therefore, it is possible that the excitation facilitates charge transfer from the aligned FF-PNT/Ag NP template to 4-ABT.

Additional evidence of charge transfer processes taking place was observed by using low concentrations (10^−9^, 10^−10^, 10^−12^, and 10^−13^ M) of 4-ABT. SERS intensity increased as the UV irradiation time increased, causing spectral features to become more visible (Fig. S13). At these concentrations, the spectrum for 4-ABT possesses features different from those observed at higher concentrations (e.g., 10^−6^ M), in agreement with what has been reported in the literature^67–69^. At concentrations greater than 10^−9^ M, the SERS spectra contained peaks at 1180, 1144, 1131, 1390, 1432, and 1590 cm^−1^. These peaks are absent in SERS spectra when using a probe molecule concentration of ≤10^−9^ M. The SERS spectra instead exhibit peaks at 974, 1185, 1233, 1342, 1368, and 1548 cm^−1^. Peaks appearing at 1233, 1342, and 1368 cm^−1^ in the spectra can be assigned to the stretching modes of the primary aromatic amines, in agreement with the literature^67–69^. The appearance of these peaks, which are called Raman forbidden peaks of 4-ABT, is further indication that UV irradiation facilitates charge transfer processes for the aligned FF-PNT/Ag NP template.

**Figure 6:**
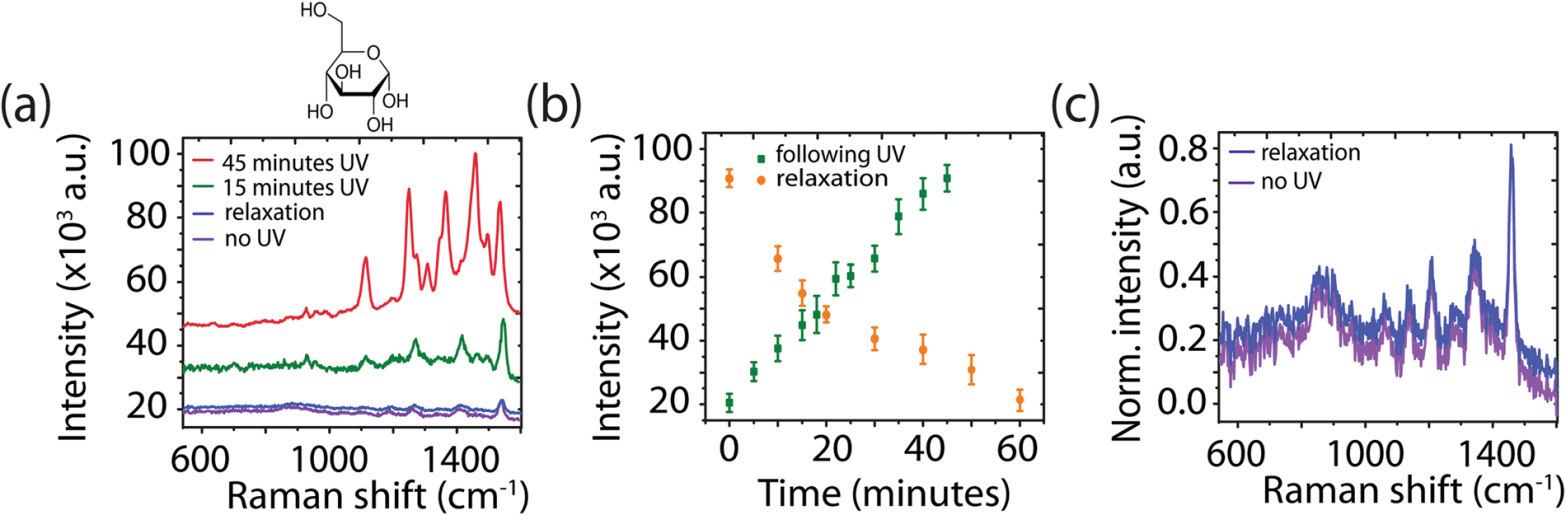
(**a**) SERS measurements of glucose on the aligned FF-PNT/Ag NP template with and without 254 nm UV irradiation. (**b**) Plot of intensity vs. time for the glucose band at 1645 cm^−1^ following 254 nm UV irradiation in 5 minute increments (green) and after the final irradiation step (orange). (**c**) Normalized SERS spectra of glucose on the aligned FF-PNT/Ag NP template without UV (purple) and after 45 minutes of total irradiation (blue).

The spectral methodology was applied to enhance the SERS signal from a biomolecule with a low Raman cross-section eg. glucose (Fig. 6a). Glucose possesses an absorption band in the UV region (<250 nm) (Fig. S2), resulting in an absence of Raman resonance enhancement. Following UV irradiation, there was significant enhancement in comparison with non-UV irradiation. SERS signal for glucose at bands 1632 cm^−1^ increased from 20 × 10^3^ to 90 × 10^3^ after a total of 45 minutes of UV irradiation (Fig. 6b). Relaxation of the Raman signal occurred over 60 minutes (Fig. 6b) as with previous probe molecules (TMPyP, RhB, and 4-ABT). The spectra obtained before UV and 60 minutes after the final 5 minute irradiation present the same bands, indicating that no measurable molecular decomposition occurred.

To demonstrate the power of combining UV irradiation with the aligned FF-PNT/Ag NP template, lower concentrations of TMPyP, RhB, and glucose (10^−13^, 10^−10^ and 10^−12^ M, respectively) were studied. The spectra presented for the three molecules are in line with it has been reported in literature (Fig. S14)^52,53,65–67^.

In summary, we present a strategy based on combining plasmonic nanoparticles with a photo-activated wide bandgap semiconductor that gives rise to an order of magnitude SERS signal enhancement for a wide range of small molecules. The thermal conductivity of the aligned peptide nanotubes further prevents photodegradation of probe molecules, likely by acting as a heat sink during UV irradiation. Charge transfer processes contribute to a greater surface-enhanced Raman intensity, allowing the detection of molecules at concentrations as low as 10^−13^ M. We propose that the mechanism for the observed chemical enhancement for aligned FF-PNT/Ag NP templates is Fermi level modification of Ag NPs through charge transfer of photogenerated electrons from FF-PNTs during UV irradiation, in conjunction with electromagnetic enhancement from Ag NPs that are densely packed between the FF-PNTs, leading to more electromagnetic hot spots. The ability to detect a variety of molecules with photodegradation-free enhanced SERS signals can lead to a wide range of applications for the aligned FF-PNT/Ag NP template. Some areas of interest are small biomolecule sensing such as glucose monitoring^22,70^.

## Experimental Details

### Preparation of FF-PNT Solution

FF-PNTs were prepared by dissolving the L-diphenylalanine peptide (Bachem, Bubendorf, Switzerland) in 1,1,1,3,3,3-hexafluoro-2-propanol (Sigma-Aldrich, Ireland) at an initial concentration of 100 mg/ml, which was then further diluted in deionized water to a final concentration of 2 mg/ml for FF-PNTs to self-assemble. Fresh stock solutions were prepared for each experiment.

### Preparation of Probe Molecule Solutions

To prepare meso-tetra (N-methyl-4-pyridyl) porphine tetrachloride (TMPyP) (T40125, Frontier Scientific) solutions, TMPyP powder was diluted with deionized water to a final concentration of 10^−4^ M. The solution was further diluted with deionized water to a range of concentrations, from 10^−5^–10^−13^ M. 4-aminothiophenol (4-ABT) (CAS 1193-02-8, New Star Chemical) 4-ABT solution was prepared by dissolving 4-ABT powder in methanol to a concentration of 10^−4^ M. The solution was then further diluted with deionized water to lower concentrations.

### UV-Vis Absorbance Spectrometer

Optical absorbance measurements of FF-PNTs with and without Ag NPs and the analyte molecule TMPyP were performed on an UV-Vis absorbance spectrometer (V-650, JASCO, Inc.) under identical settings: 1 nm step size, 1 nm bandwidth, and 400 nm/minute scan speed across a 190–900 nm range. A quartz cuvette or glass cover slip was used to conduct the measurements. Optical absorption measurements also were demonstrated on Ag NPs with and without FF-PNTs following UV irradiation at period between 5 minutes till 45 minutes, using a UV lamp with a wavelength of 254 nm and a nominal output power of 4.5 mW/cm^2^ (40–759, Edmond Optics).

### Fourier Transform Infrared Spectroscopy

FTIR measurements were performed using Alpha.Platinum-ATR (12209186, Bruker). A small drop of FF-PNT/Ag NP solution (10 μl) was placed on the ATR diamond crystal. The spectrum was collected using transmission mode scanning from 1400–4000 nm, with and without UV irradiation (254 nm) of FF-PNT/Ag NP.

### Preparation of Si Substrate

Si wafers (Si Mat), cut to 2 cm × 1 cm, were cleaned of surface contaminants by dipping in acetone for 2 minutes and then washed with ethanol and isopropanol (Sigma-Aldrich). De-ionized water was used to rinse the substrates, which were then blown dry using nitrogen. The patterned substrate was prepared as reported previously using a mask with an opening of 0.5 cm^49^.

### Preparation of FF-PNT/Ag NP Template

FF-PNT/Ag NP templates were prepared using 2 mg/ml of FF solution heated at 100 °C for 2 minutes and Ag NPs (795968, Sigma-Aldrich) with a diameter of 40 nm at a concentration of 0.02 mg/ml in water. 20 μl of Ag NP solution was added to 60 μl of the heated FF solution and stirred for 3 minutes. 30 μl of the mixed solution was then deposited on the patterned Si substrate to create the aligned FF-PNT/Ag NP template. To deposit Ag NPs on a Si substrate, 20 μl of Ag NPs (0.02 mg/ml) was diluted in 60 μl of water then 30 μl of the solution was deposited on Si substrate. Similarly, Au NPs (765546, Sigma-Aldrich) with a diameter of 40 nm at a concentration of 0.02 mg/ml in water were used to prepare aligned FF-PNT/Au NP templates.

### Optical Microscopy

Optical micrographs were used to image (10x objective) FF-PNTs bio template at different UV irradiation time between 5 minutes till 40 minutes.

### Scanning Electron Microscopy (SEM)

SEM (JSM-7600F, JEOL, operated at 5 kV) was employed to characterize and observe the location of NPs decorating the surface. A thin (~8 nm) layer of gold was sputtered on the samples before SEM imaging (Hummer IV, Anatech USA).

### Raman Spectroscopy

SERS measurements were performed using a bespoke Raman system that consisted of an inverted optical microscope (IX71, Olympus), a monochromatic laser (HeNe, ThorLabs) with beam splitter and long pass filter (RazorEdge, Semrock), a spectrograph (SP-2300i, Princeton Instruments), and a CCD camera (IXON, Andor)^49,50^. To focus the laser (532 nm wavelength, 3.56 mW incident power), a 50x objective was used. Raman spectra were collected with an exposure time of 1 s. 30 μl of the analyte molecule mol meso-tetra (N-methyl-4-pyridyl) porphine tetrachloride (TMPyP) (T40125, Frontier Scientific), rhodamine B (RhB) (132311000, Acros Organics) and 4-aminothiophenol (4-ATB) (CAS 1193-02-8, New star chemical) at different concentration starting from 10^−6^ up to 10^−9^ M was deposited above the aligned PNTs in the presence Ag NPs. The average of typically 10 measurements, is reported. Toluene was used for calibration of the Raman signal over the spectral window. SERS measurements were performed following UV lamp (254 nm) irradiation at period ranging from 5 minutes up to 45 minutes at a distance of 2.2 cm for all analyte molecules. UV irradiation occurred in 5 minute increments up to a total of 45 minutes. Raman measurements were performed when the UV lamp was off, in between each 5 minute irradiation and also over the course of 60 minutes following the final 5 minute irradiation. For reference, 30 μl of 10^−6^ M TMPyP or 4-ABT or RhB or glucose was deposited above the Si and Ag NPs on Si samples.

## Electronic supplementary material

Supplementary Information
