# Supplementary material for: Photo-induced surface-enhanced Raman spectroscopy from a diphenylalanine peptide nanotube-metal nanoparticle template
Source: Sci Rep. 2018 Mar 1;8:3880. doi: 10.1038/s41598-018-22269-x (PMC5832858; doi:10.1038/s41598-018-22269-x)
Supplement: Supplementary file 1 — Supplementary Information [file 41598_2018_22269_MOESM1_ESM.pdf]

Supplementary Information

Photo-induced surface-enhanced Raman spectroscopy from a diphenylalanine peptide nanotube-metal nanoparticle template

Sawsan Almohammed, [a, b] Fengyuan Zhang, [a, b] Brian J. Rodriguez, * [a, b] and James H. Rice * [a]

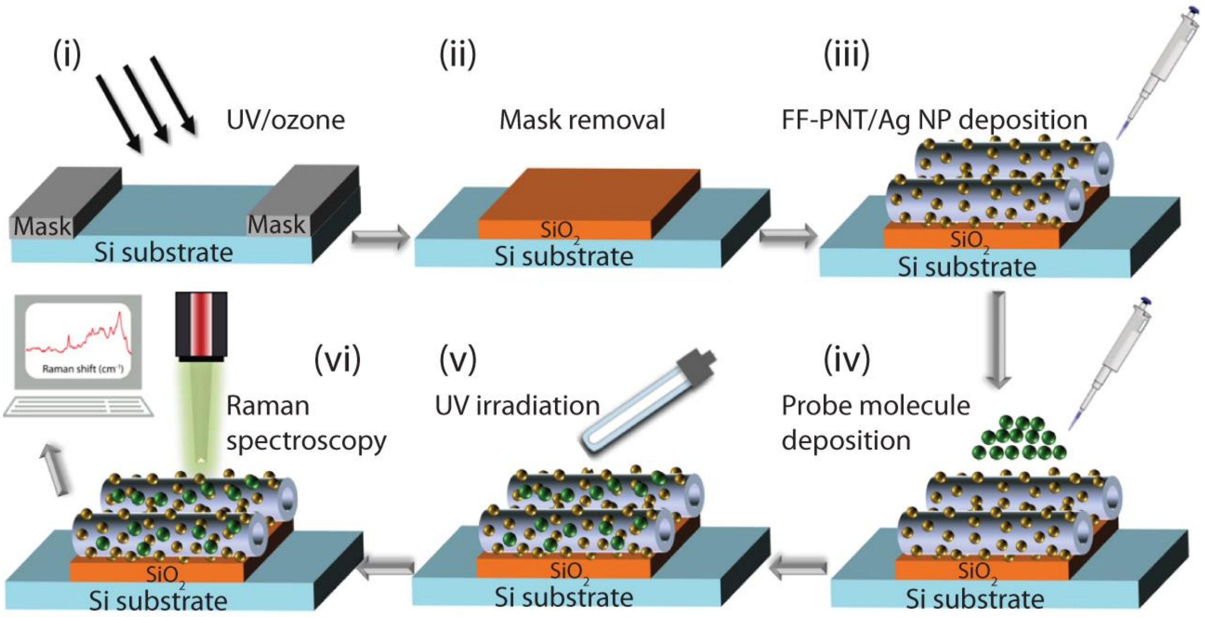

Figure S1. Schematic illustration of the preparation of the aligned FF-PNT/Ag NP template. (i) UV/ozone exposure through a 0.5 cm opening. (ii) Removal of the mask revealing hydrophobic and hydrophilic regions. (iii) FF-PNT and Ag NP deposition. (iv) Addition of the analyte molecule on the aligned FF-PNT/Ag NP template. (v) UV irradiation. (vi) SERS measurement following UV irradiation.

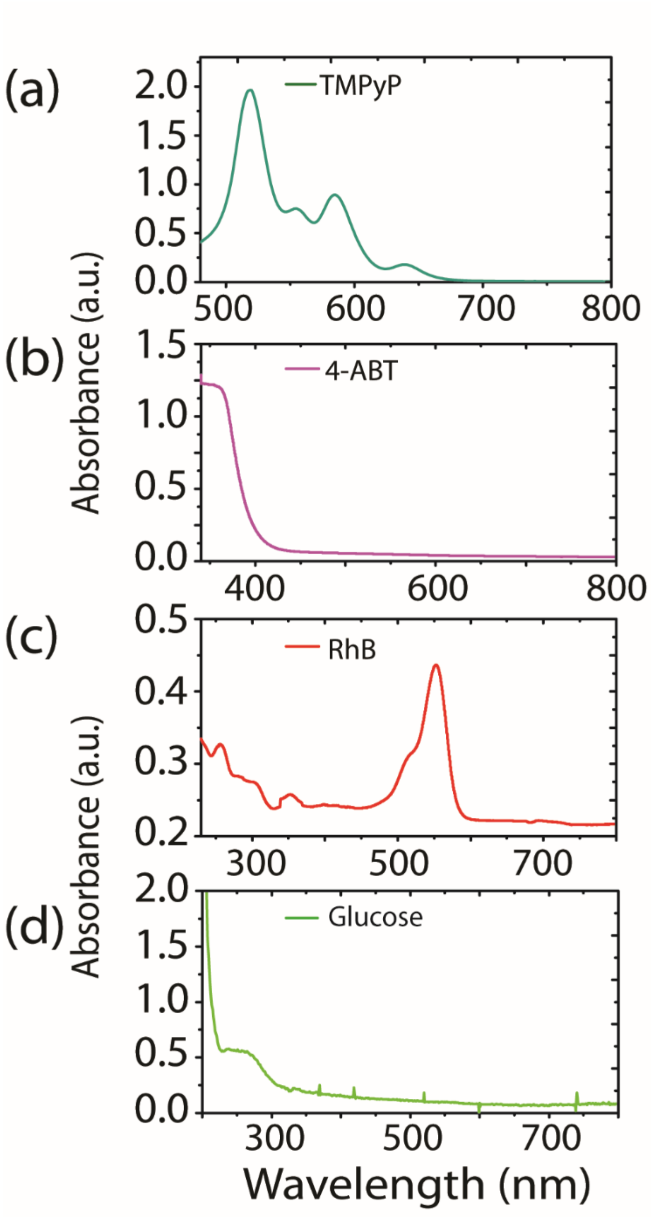

Figure S2. Optical abortion spectra of (a) TMPyP, (b) 4-ABT, (c) RhB, and (d) glucose, respectively, at concentrations of 10^{-4} M.

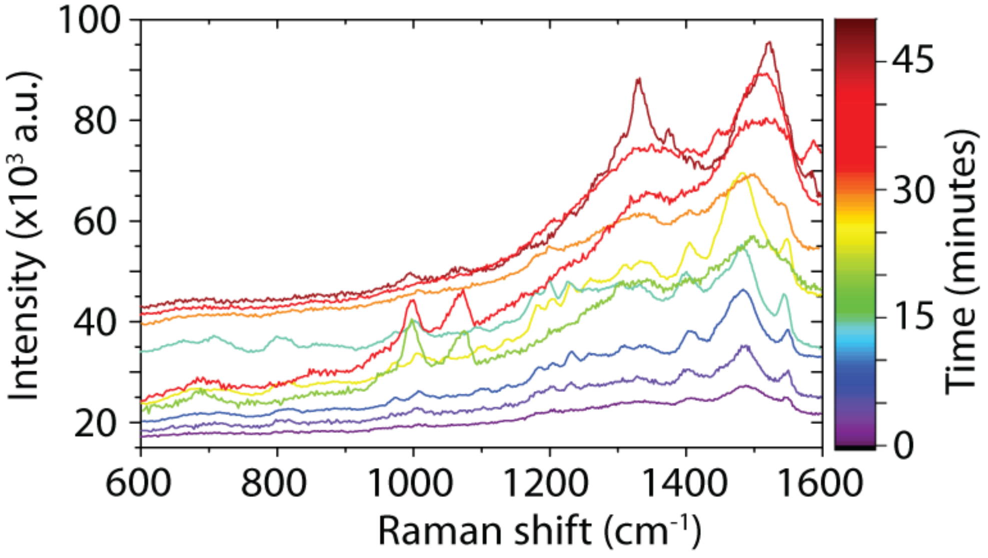

Figure S3. Series of SERRS spectra recorded from 10^{-6} M TMPyP on Ag NPs on a Si substrate before and following UV irradiation in 5 minute increments up to a total of45 minutes, showing photodegradation of the probe molecule.

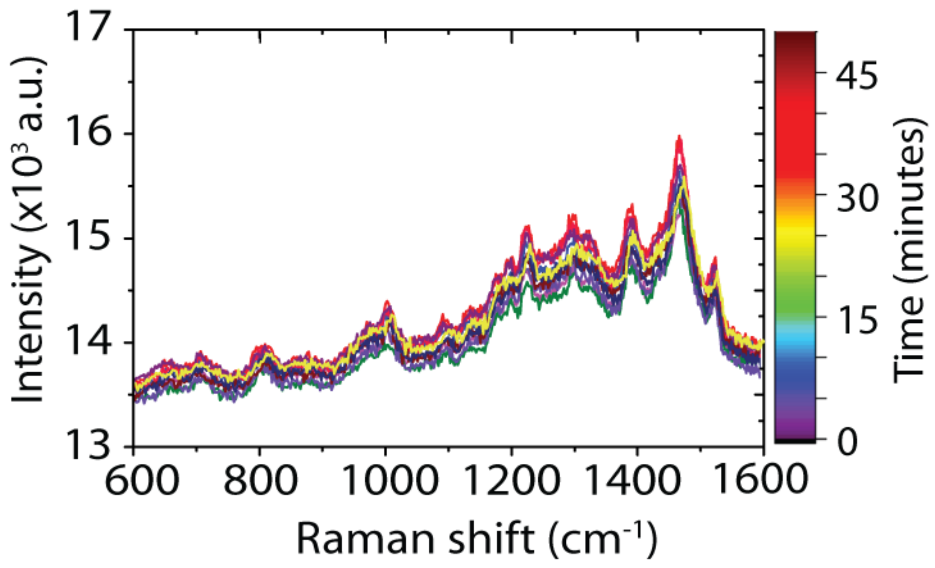

Figure S4. SERS measurements recorded from 10^{-6} M TMPyP on the aligned FF-PNT/Ag NP template before and following 365 nm UV irradiation in 5 minute increments up to a total of 45 minutes.

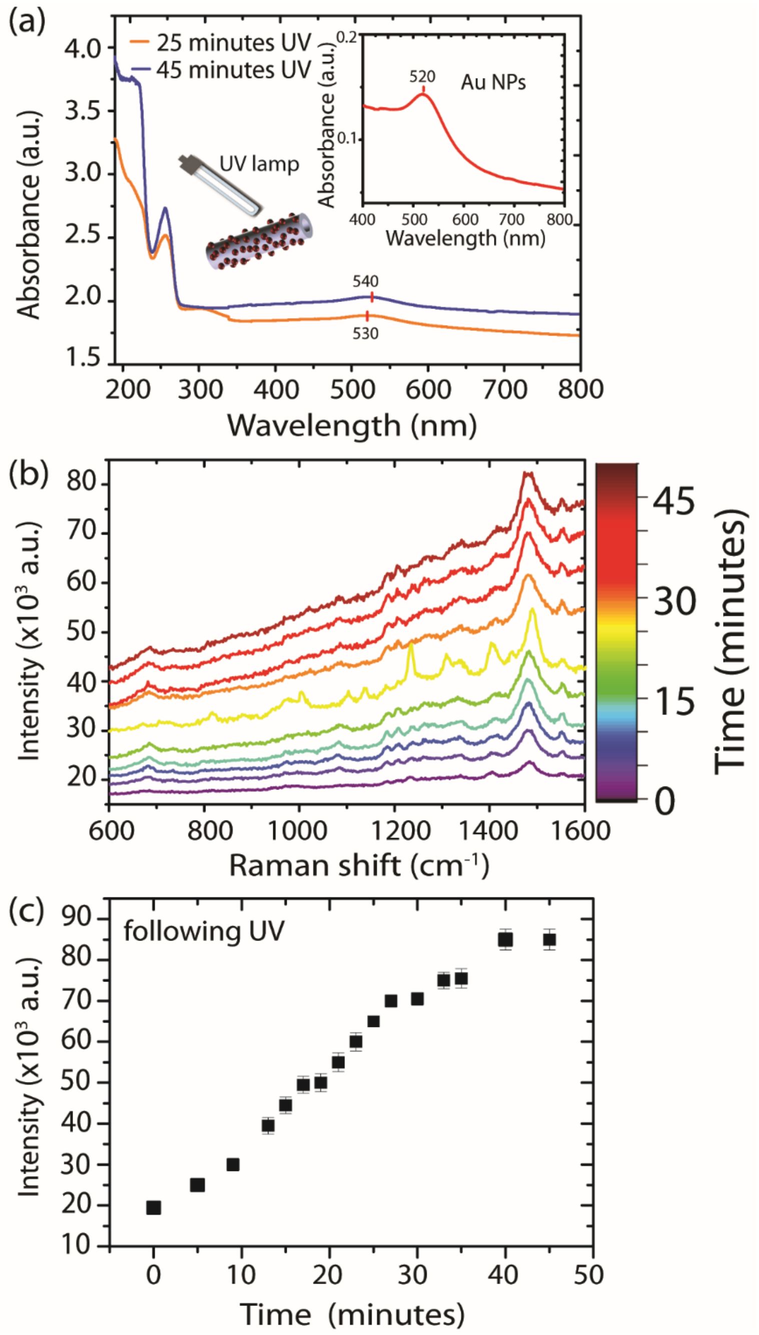

Figure S5. (a) Optical absorption of 40 nm diameter Au NPs and FF-PNTs in solution. The inset in (a) is the absorption spectrum for Au NPs only. (b) SERS measurements of 10^{-6} M TMPyP on the aligned FF-PNT/Au NP template following 254 nm UV irradiation in 5 minute increments up to a total irradiation time of 45 minutes. (c) Corresponding plot of UV irradiation time vs. intensity for the TMPyP band at 1448 cm^{-1}.

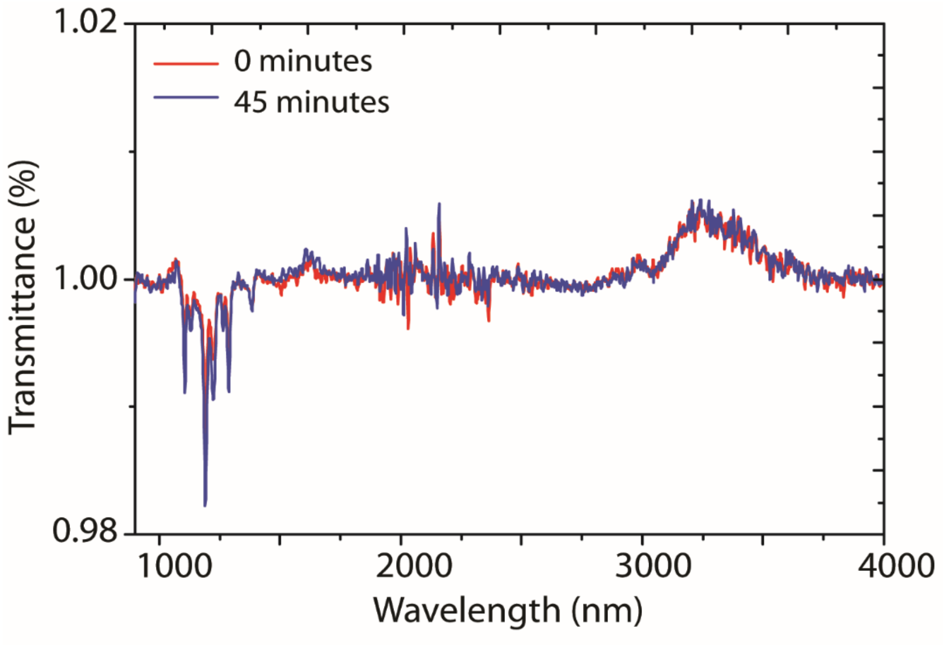

Figure S6. ATR-FTIR spectrum of FF-PNTs and Ag NPs recorded in aqueous solution. The broad nature of the amide I band at 3250 nm corresponds to β-sheet structure during peptide nanotube formation. The spectral features are similar to that reported previously for FF-PNTs.[72]

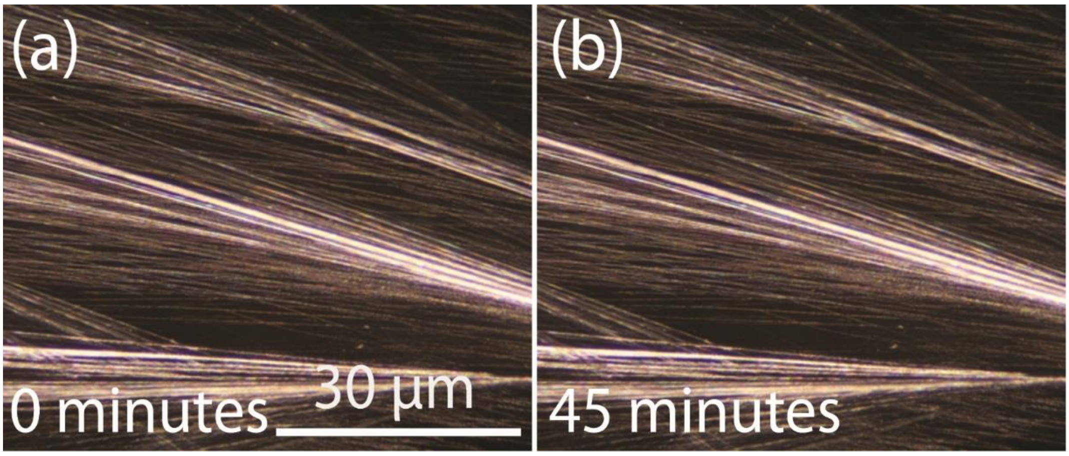

Figure S7. Optical images of aligned FF-PNT/Ag NP templates (a) before UV irradiation (0 minutes) and (b) after 45 minutes of 254 nm UV irradiation.

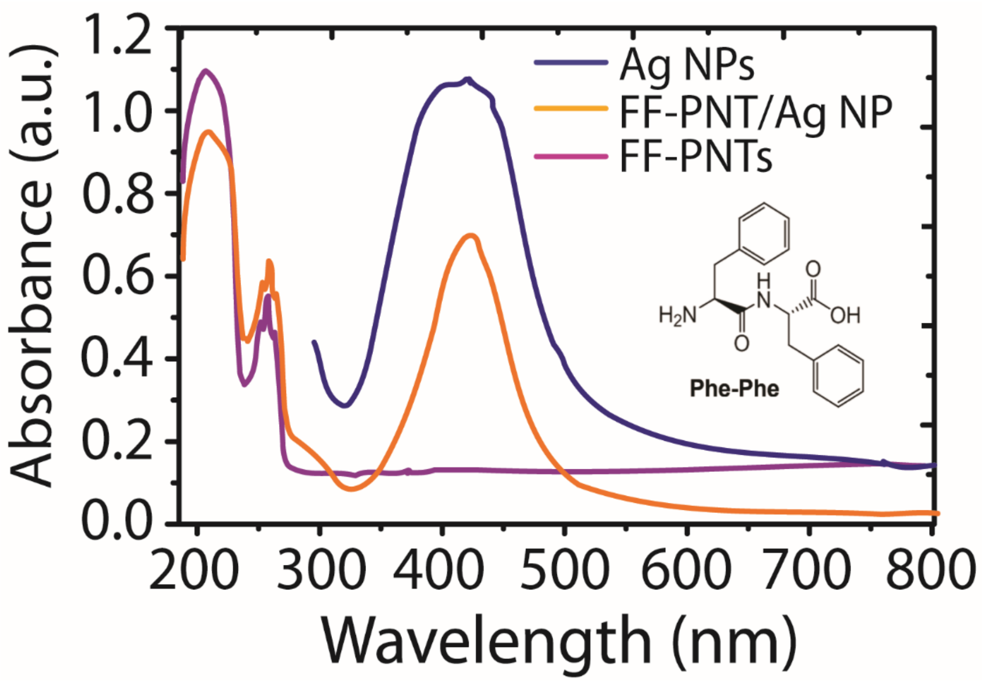

Figure S8. Absorption spectra of solutions of Ag NPs (green), FF-PNTs (blue), and FF-PNTs and Ag NPs together (purple).

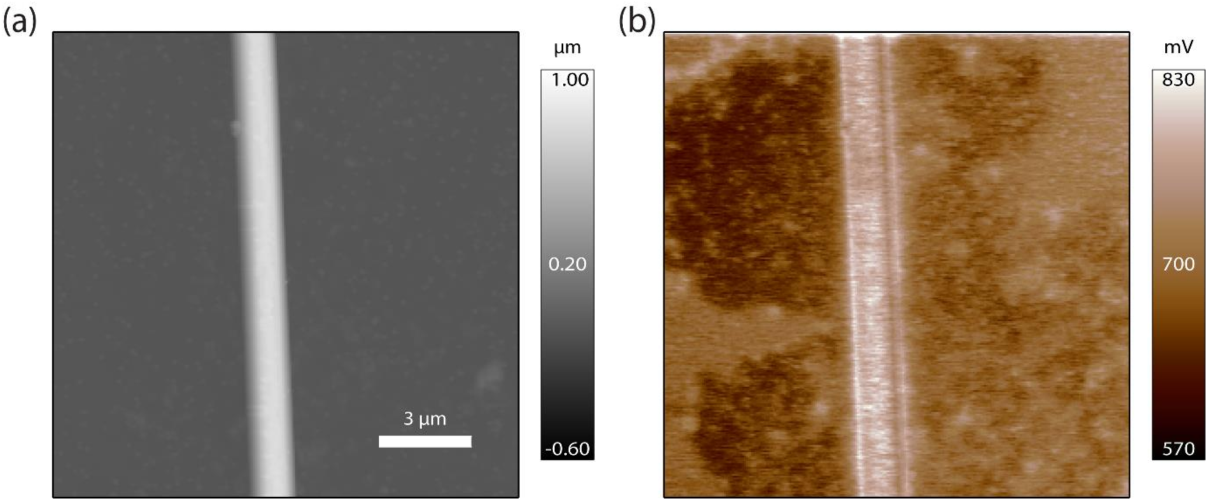

Figure S9. (a) Atomic force microscopy (AFM) height image of a FF-PNT and (b) Kelvin probe force microscopy (KPFM) potential image of the same FF-PNT showing the contact potential difference between the Ir-coated tip (ASYELEC-01, Asylum Research) and the surface. The CPD between the tip and the FF-PNT was determined using a mask to be 786.7 ± 17.2 mV. Assuming Ir has a workfunction of 5.5 eV,[1] the workfunction of the FF-PNT is determined to be ~ 6.3 eV. KPFM was performed using an Asylum Research MFP-3D AFM and a lift height of 40 nm.

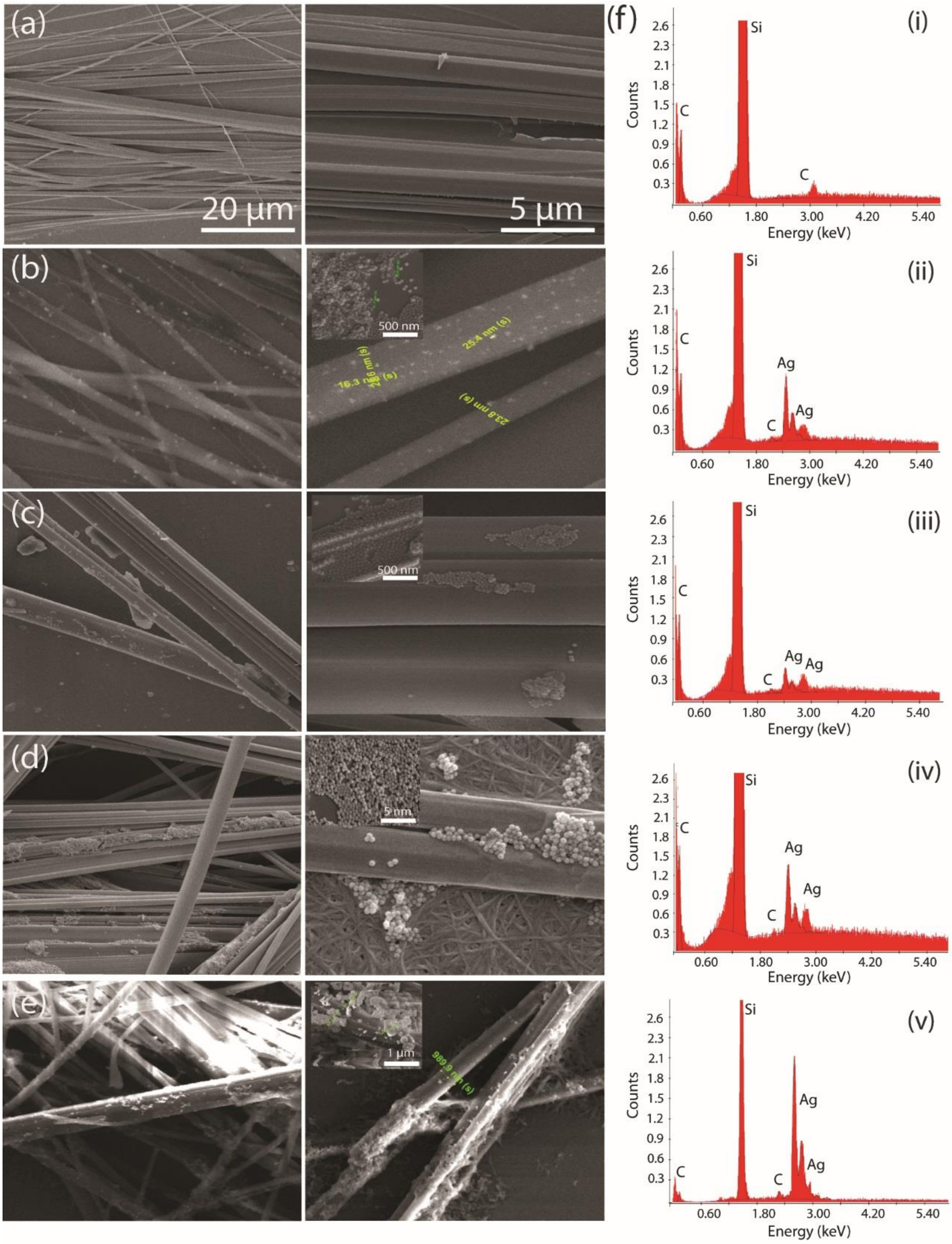

Figure S10. SEM images of (a) FF-PNTs and (b-e) FF-PNTs with photoreduced Ag NPs (0.01 M $AgNO_3$) following 10, 15, 20, and 30 minutes of UV irradiation, respectively. The same UV lamp was used for the Raman measurements. The size of the NPs, as determined from SEM images (n = 20 NPs), increased with increasing exposure time: 21.83 ± 3.01 nm, 33.46 ± 2.70 nm, 46.96 ± 3.51 nm, 165.61 ± 3.87 nm for 10, 15, 20, and 30 minutes of UV irradiation, respectively. (f) Energy dispersive X-ray of (i) FF-PNTs without NPs shows the absence of Ag whereas (ii), (iii), (iv), and (v) show the presence of Ag on samples shown in (b), (c), (d), and (e), respectively.

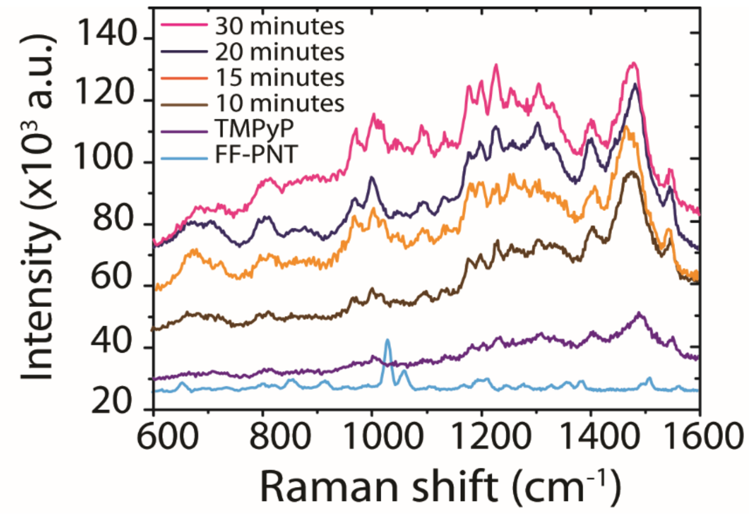

Figure S11. SERS measurements of 10^{-5} M TMPyP on an aligned FF-PNT/Ag NP template prepared via photoreduction of Ag NPs from 0.01 M $AgNO_3$ solution during 10, 15, 20, and 30 minutes of UV irradiation. The blue spectrum was recorded from aligned FF-PNTs in the absence of NPs and analyte. The purple spectrum was recorded from 30 µl of 10^{-5} M TMPyP deposited on a Si substrate.

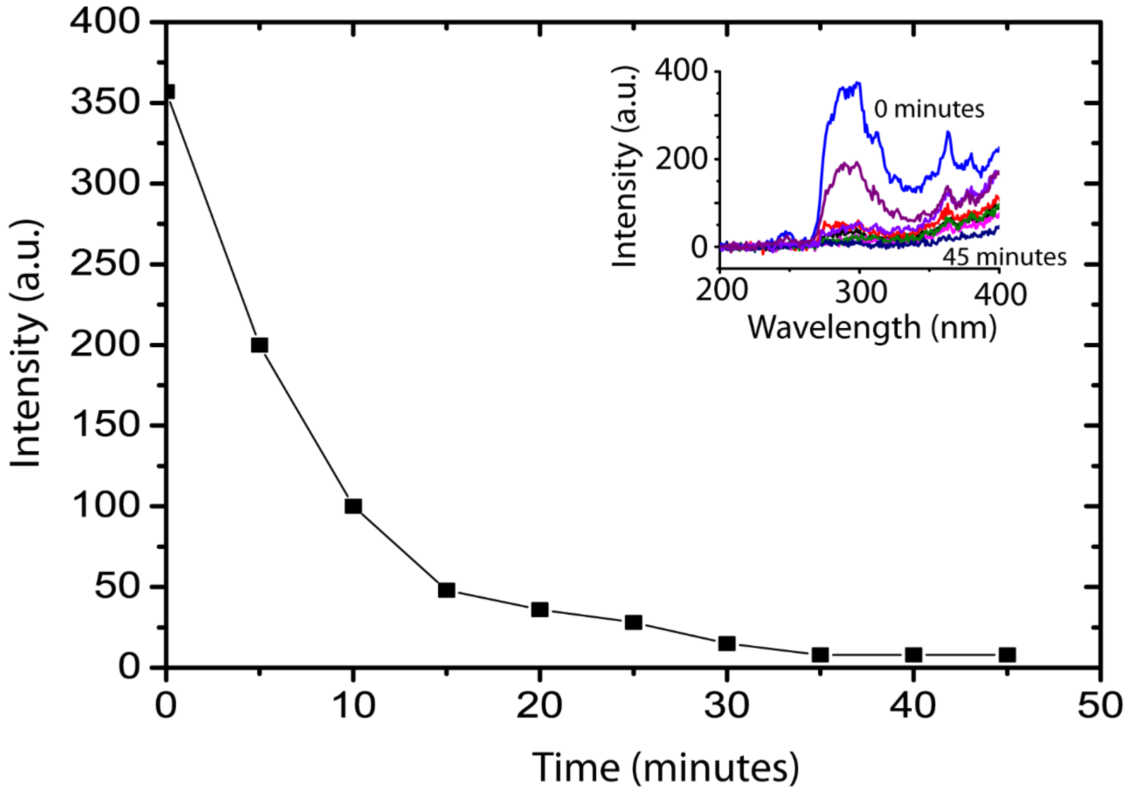

Figure S12. Plot of photoluminescence intensity at 300 nm emission peak measured from an aligned FF-PNT/Ag NP template on a cover slip showing a decrease in intensity with increasing UV irradiation time. Inset shows the photoluminescence spectra. The measurements were performed using a Cary Eclipse Fluorescence Spectrophotometer (Agilent Technologies).

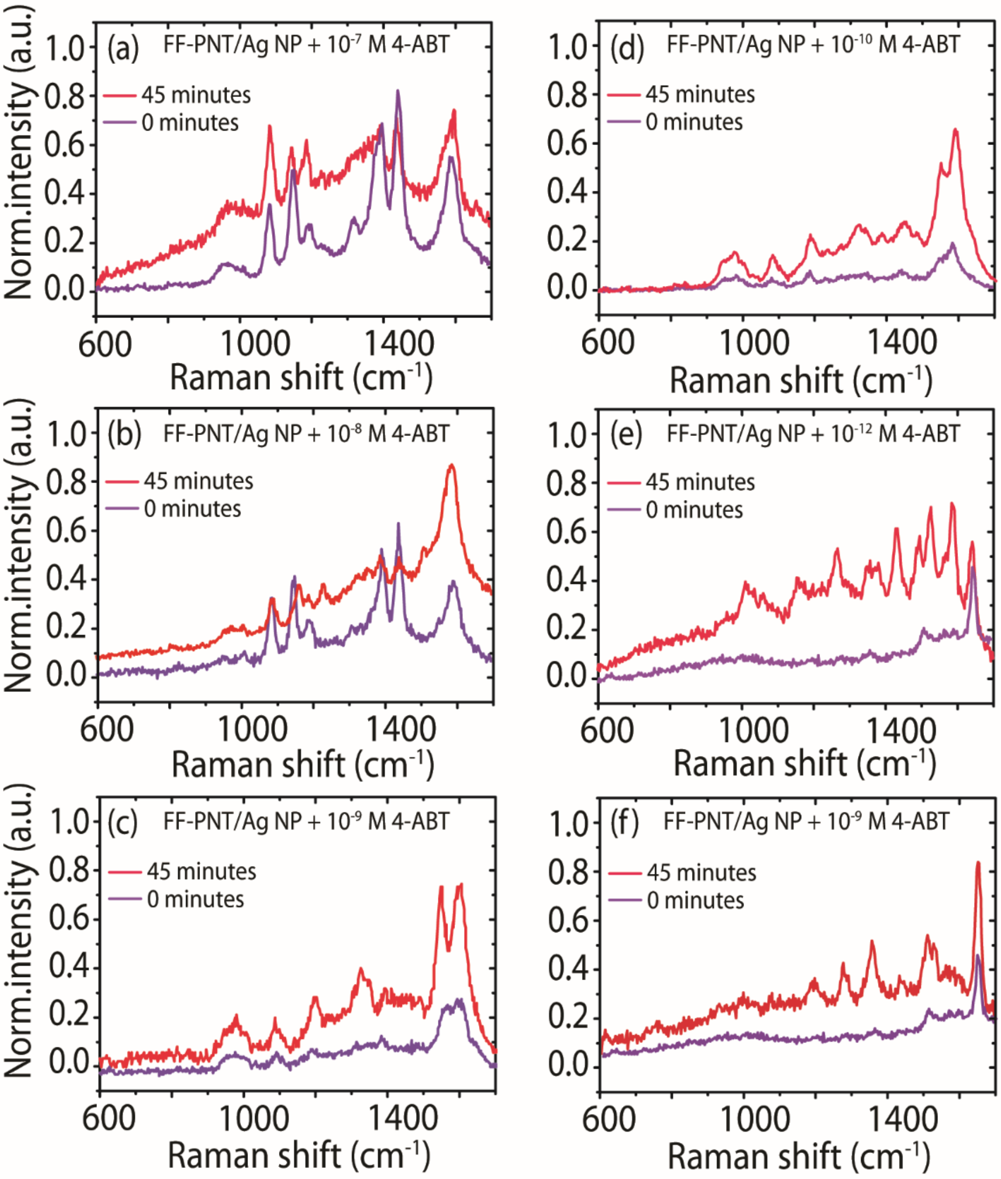

Figure S13. SERS measurements of the aligned FF-PNT/Ag NP template and the probe molecule 4-ABT at concentrations of (a) 10^{-7} M, (b) 10^{-8} M, (c) 10^{-9} M, (d) 10^{-10} M, (e) 10^{-12} M, and (f) 10^{-13} M before (purple) and after (red) a total of 45 minutes of 254 nm UV irradiation.

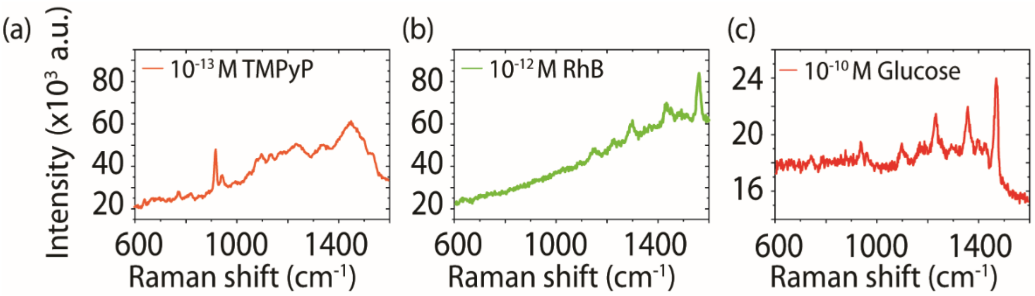

Figure S14. SER(R)S measurements of aligned FF-PNT/Ag NP templates with (a) 10^{-13} M TMPyP, (b) 10^{-12} M RhB, and (c) 10^{-10} M glucose following 45 minutes of 254 nm UV irradiation.

References

1. Reddy, B. N., Kumar, P. N. & Deepa, M. A poly(3,4-ethylenedioxypyrrole)–Au@WO_3-based electrochromic pseudocapacitor. ChemPhysChem **16**, 377–389 (2015).
